# Rehabilitation needs for older adults with stroke living at home: perceptions of four populations

**DOI:** 10.1186/1471-2318-7-20

**Published:** 2007-08-13

**Authors:** Claude Vincent, Isabelle Deaudelin, Line Robichaud, Jacqueline Rousseau, Chantal Viscogliosi, Lise R Talbot, Johanne Desrosiers, other members of the BRAD group

**Affiliations:** 1 Centre for Interdisciplinary Research in Rehabilitation and Social Integration (CIRRIS), Institut de réadaptation en déficience physique de Québec, 525, Wilfrid-Hamel Blvd East, Québec (Québec), G1M 2S8, Canada; 2 Département de réadaptation, Université Laval, Pavillon Ferdinand-Vandry, Québec (Québec), G1K 7P4, Canada; 3 École de réadaptation, Université de Montréal, Pavillon Marguerite D'Youville, c.p. 6128, succursale Centre-ville, Montréal (Québec), H3C 3J7, Canada; 4 Research Centre on Aging, University Institute of Geriatrics of Sherbrooke, 1036 Belvédère South, Sherbrooke (Québec), J1H 4C4, Canada; 5 Department of Nursing, Faculty of Medicine and Health Sciences, Université de Sherbrooke, 3001, 1^st ^Avenue, Sherbrooke (Québec), Canada; 6 Department of Rehabilitation, Faculty of Medicine and Health Sciences, Université de Sherbrooke, 3001, 12^th ^Avenue, Sherbrooke (Québec), Canada; 7 Research Center on Aging, University Institute of Geriatrics of Montreal, 4564 Queen Mary Road, Montréal (Québec), H3W 1W5, Canada

## Abstract

**Background:**

Many people who have suffered a stroke require rehabilitation to help them resume their previous activities and roles in their own environment, but only some of them receive inpatient or even outpatient rehabilitation services. Partial and unmet rehabilitation needs may ultimately lead to a loss of functional autonomy, which increases utilization of health services, number of hospitalizations and early institutionalization, leading to a significant psychological and financial burden on the patients, their families and the health care system. The aim of this study was to explore partially met and unmet rehabilitation needs of older adults who had suffered a stroke and who live in the community. The emphasis was put on needs that act as obstacles to social participation in terms of personal factors, environmental factors and life habits, from the point of view of four target populations.

**Methods:**

Using the focus group technique, we met four types of experts living in three geographic areas of the province of Québec (Canada): older people with stroke, caregivers, health professionals and health care managers, for a total of 12 groups and 72 participants. The audio recordings of the meetings were transcribed and NVivo software was used to manage the data. The process of reducing, categorizing and analyzing the data was conducted using themes from the Disability Creation Process model.

**Results:**

Rehabilitation needs persist for nine capabilities (e.g. related to behaviour or motor activities), nine factors related to the environment (e.g. type of teaching, adaptation and rehabilitation) and 11 life habits (e.g. nutrition, interpersonal relationships). The caregivers and health professionals identified more unmet needs and insisted on an individualized rehabilitation. Older people with stroke and the health care managers had a more global view of rehabilitation needs and emphasized the availability of resources.

**Conclusion:**

Better knowledge of partially met or unmet rehabilitation needs expressed by the different types of people involved should lead to increased attention being paid to education for caregivers, orientation of caregivers towards resources in the community, and follow-up of patients' needs in terms of adjustment and rehabilitation, whether for improving their skills or for carrying out their activities of daily living.

## Background

Stroke is the third leading cause of long-term disability [1] and its incidence increases markedly with advancing age [2]. With improvements in health care, more people survive strokes but many have to cope with the physical, psychological, social and functional sequelae, resulting in increased personal and public costs [1,3-5] and a marked decline in their quality of life [6-11]. After a stroke, most elderly people return to their home environment quickly, despite suffering from various impairments and disabilities and often without having received any rehabilitation services to reduce or compensate them [12,13]. In Canada, only about 10 to 15% of people with stroke receive inpatient rehabilitation services [14]. The other survivors, whose physical deficits are not so severe or whose impairments and disabilities are not properly identified, return to their own environment, with or without support services [15,16]. In order to better plan the offer and delivery of rehabilitation services, the partially met or unmet needs of people who live at home after stroke are little known, especially from the perspective of the different actors involved in this process. Even though many studies have been carried out on recovery from impairments and disabilities after stroke and the consequences of such disabilities, very few are interested in exploring partially met and unmet rehabilitation needs that could restrict the social participation of these people in their daily activities and social roles.

The aim of this study was to explore partially met and unmet needs of adults aged 65 years or over who had suffered a stroke and who live in the community, with or without services. The emphasis was put on the needs that are considered obstacles to social participation from the point of view of older adults with stroke, caregivers, health professionals and health care managers.

## Literature review

What do we know about social participation and rehabilitation needs for people with stroke?

### Social participation

**Figure 1:**
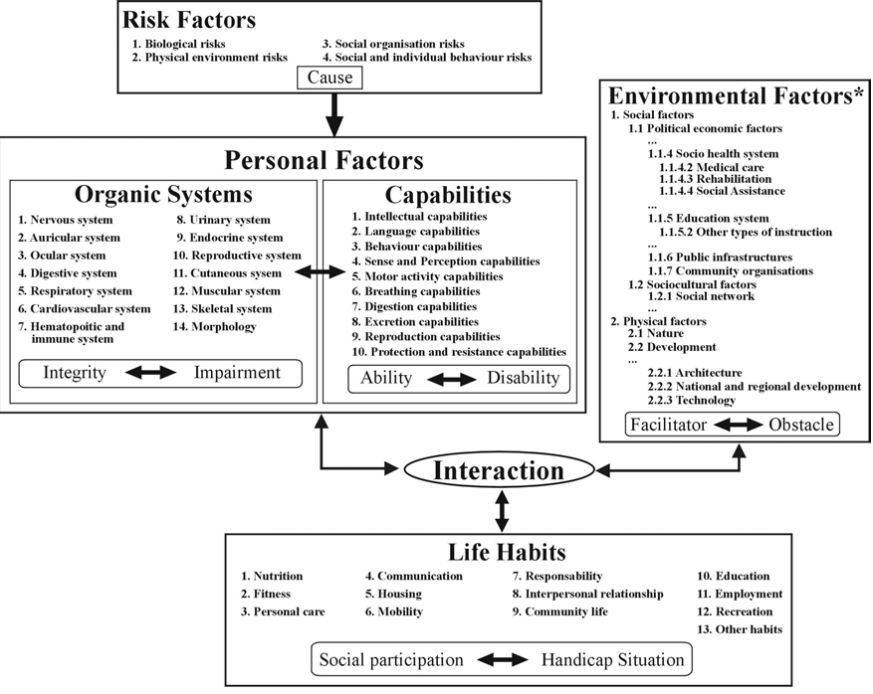
The Disability Creation Process, conceptual scheme (Fougeyrollas, Cloutier, Bergeron, Côté, Côté & St-Michel, 1998). CRIPPH 1998. ripph@irdpq.qc.ca. 1(418)529-9141, p.6202. *Authors of this manuscript have added subcategories to the original scheme to facilitate the understanding of text.

According to the Disability Creation Process (DCP) model, social participation or its opposite, handicap situation, is identified as a situational result that varies over time depending on the interaction between personal factors (the individual's organic system, capabilities, identity) and environmental factors (social and physical) [17,18] (Figure 1). This systemic model of human development, which is widely known and used in Canada, is based on the interaction between individuals and their environment. This model is very useful for identifying and classifying variables under study with greater systematization and consistency.

Stroke highly affects **personal factors **that include both impairment and disability. There is major impairment of the nervous system and other organic systems (e.g., muscular, ocular). The consequences of these impairments are operationalized through the presence of disabilities in capabilities (e.g., intellectual, language, behaviour, sense and perception, motricity and balance). Depending on the number and extent of these disabilities, people vary in their ability to accomplish daily activities and social roles (presence of handicap situations at one end of the spectrum and optimal social participation at the other) [19].

The characteristics of the person's environment also affect social participation after stroke. Social **environmental factors **include elements such as the support from the family and utilization of health and social services, whereas physical environmental factors refer to natural or technological elements (e.g., climate, technical aids). As obstacles or facilitators, these factors can either hinder or help in the accomplishment of daily activities and social roles.

Finally, the different activities and roles that the person values (called '**life habits' **in the model) are divided into 12 domains. Six of these domains refer to the person's daily activities (nutrition, fitness, personal care, communication, housing, mobility) whereas the other six refer to social roles (responsibility, interpersonal relationships, community life, education, employment, recreation). It is in these activities and roles that handicap situations or restriction of participation can arise. In the DCP model, needs might be considered as the outcome of a lack of congruence between personal factors and different factors in the environment [18].

Because of the high incidence of stroke [20] and its potentially negative impact on various aspects of a patient's life [5,10,21-23], studies on social participation were carried out among people who had suffered a stroke. The results of a telephone survey conducted by Mayo et al. in 2002 [24] among 434 respondents show that participation by this clientele in basic activities such as eating, dressing and moving around is less often restricted (39%) than participation in domestic tasks such as going shopping and cleaning the house (54%) which, in turn, is less affected than participation in community activities (65%) associated with social roles.

### Rehabilitation needs

Specific studies on rehabilitation needs are still rare [15,22,25-28], which means that generic literature on needs is applied to rehabilitation. Bradshaw's taxonomy of social need [29], as used by Pineault and Daveluy [30], identifies four types of needs: felt, expressed, normative and comparative. *Felt *needs are equated with wants and are limited by the perceptions of the individuals in regard to the health services available [29]. *Expressed *needs are demands or felt needs turned into action. They are commonly used in health care services where waiting lists are taken as a measure of unmet need [29]. *Normative *needs are those defined by health professionals, administrators or experts in relation to norms or a desirable standard [29]. Finally, *comparative *needs refer to a measure established by studying the characteristics of those in receipt of a service, in other words, populations in which the evaluated needs are generalized [29].

The goals of rehabilitation services and programs are currently based on the judgment of professionals who evaluate functional disabilities, ability to carry out daily activities and significant leisure, occupational and social activities, as well as health problems [31], to determine needs. These needs assessments are often incomplete because assessments are done only once, most often with standardized tests and outside the person's home environment or community context [15,16,32]. Furthermore, although family members may have more difficulty identifying needs than the patients themselves and the professionals [33], their input is essential to a needs assessment [16,34,35]. Consideration both of the needs expressed by individuals and their caregivers, and of the normative needs identified by health professionals, ensures a more reliable result, since many patients can be more passive than expected in expressing their needs [25,32,36-39]. Also, expressed needs for rehabilitation services may greatly vary from one area to another [27,40-42], possibly because of differences in accessibility. In addition, repeated measures post-stroke show that needs change over time [14,28,38,43].

Based on the DCP model, there are needs related to personal factors (capabilities) [9]. Indeed, motor and sensory problems on the side contralateral to the brain lesion [12,14,16,44,45] as well as perceptual and cognitive [14,47-51] and psychosocial [14,40,46,49-59] disorders, disrupt the daily lives of people with stroke. Often, older adults with stroke do not spontaneously find effective strategies to cope with the affective (apathy, depression, emotional variation) and cognitive problems (memory, attention, concentration, organization, judgment, communication) [60-62]. Even after adjusting for the degree of physical disability, people with cognitive deficits remain more dependent, and this dependence has increased two years after the stroke [49]. In a study carried out in Denmark, the patients reported the need for supervision and advice to continue proper physical and cognitive training at home [63]. Martin et al. (2002) [19] and Pierce et al. (2004) [64] presented similar outcomes in their recent works.

Some needs related to environmental factors are documented in literature but most of them refer mainly to rehabilitation services and education, without considering other important environmental elements. Following inpatient rehabilitation, people who had suffered a stroke expressed unmet needs related to preparation for discharge, instructions, information and support with referrals to community resources, rehabilitation services, exercise programs at home, support in doing exercise programs, support for the individual and couple during the adaptation process, nutrition, safety and housework [55,60,65-68]. The optimum benefit from rehabilitation is often not achieved during hospitalization, because of the stress experienced in the acute phase [11,59]. Thus, there is a perceived lack of care continuity [23,41,69]; one of the aims of the single entry point, which is currently being implemented in some regions, is to fill this gap [70]. During the rehabilitation process, some individuals progress more slowly and may need treatment to improve recovery for up to two years post-stroke [43,71,72]. Education, combined with counselling for self-assessment, could help people become more aware of their needs, which would help them adjust better to their disabilities [27,73]. Rehabilitation should focus more on satisfaction with life and leisure activities than simply on independence in day-to-day activities [5,32,62]. Brandriet and colleagues [60] studied perceived needs post-stroke after discharge from inpatient rehabilitation among a small number of individuals (n = 20) in a single metropolitan area; post-stroke survivors indicated they needed more therapy (physical therapy, occupational therapy and speech therapy) whereas they and caregivers also reported the need for greater social support. The recent focus group study of Hare et al. [72] combined two groups of experts (27 patients and 6 caregivers). The study concluded that better methods were required for providing information to long-term survivors of stroke and for addressing their emotional and psychological needs [22,46]. As mentioned earlier, literature concerning other types of needs related to environmental factors is limited. In one example, Evans and Northwood [73] carried out a study among a heterogeneous population aged 43 to 87 years; one of their conclusions was that there exist social assistance needs for adjustment to stroke.

For needs related to life habits, the third component of the DCP model, most of the previous studies point out general problems with mobility and instrumental activities of daily life [46,60-62,74]. In the Brandriet et al.'s study [60], post-stroke survivors indicated specific unmet needs for housekeeping, financial, nutritional and safety aspects as well as for relearning skills (maintenance/household tasks). Between one and four years post-stroke, balance, walking and instrumental activities of daily life such as personal care, housekeeping, cooking and psychosocial activities have deteriorated [11,75].

The present group of authors had previously carried out a preliminary study with four different small groups of experts (patients, caregivers, health professionals and health care managers) from a single semi-urban area [76]. Results showed partial and unmet needs relating to personal factors (mainly capabilities), environmental factors and life habits. Analysis of environmental factors also revealed the need for social support, the need for more rehabilitation services and the importance of the caregiver's role. Results were sufficiently relevant to motivate a more in-depth study in different socio-geographic areas including urban and rural areas.

In summary, context of all of the above studies was restricted, which limits their external validity. Most literature considers capabilities, the socio-health system, the education system and some life habits (daily activities); there is very little information about unmet rehabilitation needs related to social roles and environmental factors such as social assistance, public infrastructures, community organizations, social network and physical factors. Also, most of the literature refers to normative needs (from the health professionals' perspective). In 2002, when our study began, very few studies put emphasis on expressed needs from the standpoint of older adults with stroke and caregivers. After 2004, studies were published on that subject [32,34,37,38,77,82]. We could not find any literature written from a health care manager's perspective except for Talbot et al. [76], our pilot study. Since most people report a reduction in their activities and interests post-stroke [74], stroke has a substantial impact on the accomplishment of daily activities and social roles (life habits) that are essential to well-being and personal development, even when impairment and disability are mild [10].

## Objective of the study

This study examined partially met and unmet rehabilitation needs (expressed and normative) for people with stroke aged 65 and over, with respect to their personal factors (capabilities) and life habits, and environmental factors, with a view to maintaining optimal social participation. Better knowledge and understanding of rehabilitation needs and services for this population should contribute to changes in clinical and organizational practices.

## Methods

### Design

The study, conducted in 2005, followed a cross-sectional design using the focus group technique [78], individual interviews and a qualitative analysis strategy [79,80].

### Sample and eligibility criteria

To enhance the transferability [80,81] of results, this study was conducted in three regions of the province of Quebec: Montreal (metropolitan area : 1 873813 people, 3671,3 per km^2^, 15% over 65 years old), Eastern Townships: (rural area : 300 383 people, 29,5 per km^2^, 14,4% over 65 years old) and Chaudière-Appalaches (rural area: 396171 people, 26,3 per km^2^, 13.8% over 65 years old). In Canada (30000000 people), there are over 50,000 strokes each year – including 16,000 deaths. Four target populations were identified: 1) older people with stroke, hereinafter called 'patients', 2) family caregivers, 3) health professionals, and 4) health care managers involved in the rehabilitation of older adults. Caregivers and stroke survivors were independent of each others. The patients and family caregivers were recruited through support groups for people who had suffered a stroke, such as stroke clubs or community home services centres. Specific criteria (theoretical sampling) were defined, such as various levels of disabilities, gender, and different ages, in order to capture different experiences. The health professionals and the health care managers came from various clinical, institutional and community environments providing rehabilitation services to older adults. The health professionals were recruited through professional service coordinators, who were contacted by phone after receiving a letter explaining the research project. The health care managers were recruited through the regional health and social service boards in the three study regions.

For the eligibility criteria, the patients had to: a) have had at least one stroke, with the most recent occurring after the age of 65 and the previous at least two years earlier b) be living at home, and c) be able to verbally express their needs. Three quarters of people who had a stroke are aged over 65 years and, in Canada, specific health care programs are offered to this clientele, justifying the age criterion. We excluded people affected by other limitative neurological, sensory, musculoskeletal or chronic diseases, to avoid confusion between the needs related to these diseases and the needs associated with stroke. The caregivers had to: a) be the informal (family) caregiver for at least one year of a person with stroke who meets the above criteria, b) provide at least two hours of help per week, and c) be 18 years or older. The health professionals had to have at least two years' experience in an environment providing services to older adults with stroke. Finally, the health care managers had to come from the rehabilitation settings specified above. All participants should have been able to express themselves in French.

### Focus group technique and individual interviews

Focus groups were organized in each region, with patients, family caregivers, health professionals and health care managers, for a total of 12 groups (4 populations × 3 regions). The meetings lasted approximately two hours each and were recorded on audiotape. A moderator, an assistant moderator and an observer (researcher) were present at each group meeting. The same research team attended the meetings in all three regions. Focus group interview guides, refined after the pilot study, are presented in Talbot et al.'s paper [76]. To ensure the group meetings ran smoothly and generated the best content, 4–5 participants were sought for each group of patients and caregivers, and 7–8 participants were expected for each group of health professionals and health care managers [78].

In addition, to capture a wider variety of experience related to the needs expressed, 4 individual interviews with patients and caregivers were conducted, since some participants were not able to participate in the focus group meetings for various reasons (reduced mobility, uncomfortable in a group situation, caregiver unavailable).

### Data analysis strategy and conceptual framework

The focus group participants' profile was described in terms of age, gender and other relevant characteristics, depending on the group. The audiotape of the content of each group (n = 12) and during interviews (n = 4) was transcribed, then coded, recoded and classified. NVivo software (v.2) was used to manage the data. The analysis process was systematic and rigorous and respected scientific criteria for qualitative research [79,80]. An initial classification of the data was based on the Disability Creation Process presented earlier. All the transcriptions were recoded by the groups' researchers to validate the process of coding, i.e. the accuracy of the theoretical classification and the emerging classification themes. Disagreements were discussed (30% of the codification) and decisions reached by consensus to finally converge on emerging themes that made sense and which accurately reflected the content of the discussions. Data were analyzed considering two aspects: 1) For each type of group, we calculated iterations for each theoretical theme (the number of times a participant discussed a specific theme); 2) For the content, we created emerging themes reflecting the discussions and classified them under the theoretical aspect.

## Results

### Participants' profile

In all three regions, 17 persons with stroke and living at home took part in individual interviews (n = 3) and focussed discussion groups (n = 14) (Table 1). Their age varied between 65 and 85 years. Most had had their stroke over two years previously and were living alone or with their spouse. Patient's profile meets the specific criteria "various levels of disability representation" because less than one third were receiving home care assistance from the public health care system, while over half were being cared for informally by their next of kind. Also, three patients with more severe limitations were met at home. The patients' educational level varied.

**Table 1:** Description of characteristics of people with stroke

**Patients (N = 17)**	**Eastern Townships (n = 8)^1^**	**Montreal (n = 3)**	**Chaudières-Appalaches (n = 6)**
**Age**:			
65–75 years	4	1	5
76–85 years	4	2	1

**Gender (M)**	5	0	5

**Living environment (urban)**	3	3	3

**Time elapsed since stroke**:			
< 1 to 3 years	4	1	4
4–8 years	4	1	2
9 years and +	0	1	0

**Education level**:			
Elementary	6	1	2
Secondary	2	1	3
Post-secondary	0	1	1

**Registered for public home care services**	2	1	2

**Receive assistance from relatives or friends**	5	3	3

Note 1 In this region, 3 patients were interviewed individually.

Twelve close caregivers aged between 41 and 69 participated in focussed discussion groups (n = 11) and one individual interview (n = 1) (Table 2). In general, the caregivers interviewed were assisting people who were more severely disabled than the patients recruited to participate in the focus groups for persons with stroke. They were either the spouse or the daughter of the person for whom they had been caring for at least one year, from two to twenty hours per week in various ways. Most of the caregivers were retired.

**Table 2:** Description of characteristics of close caregivers

**Caregivers (N = 12)**	**Eastern Townships (n = 6)^1^**	**Montreal (n = 3)**	**Chaudières-Appalaches (n = 3)**
**Age**:			
41–59 years	4	1	0
60–69 years	2	2	3

**Gender (F)**	5	3	1

**Living environment (urban)**	2	3	2

**Current occupation**:			
Works outside the home	2	2	1
Retired	4	1	2

**Relationship with patient**:			3
Spouse	5	2	
Daughter	1	1	0

**Experience as a caregiver:**			
1 to 4 years	3	2	2
over 4 years	3	1	1

**Weekly assistance**			
0–12 hours	3	2	2
13–20 hours	3	1	0
over 20 hours	0	0	1

**Type of assistance given**:.			
A.D.L	5	3	2
I.A.D.L.	6	3	3
Psychological support	4	3	2
Other(stimulation)	3	3	0

In this group of respondents, only one caregiver was interviewed individually.

Twenty-five health professionals participated in the discussion groups (Table 3). They had more than nine years of clinical experience. They were working in six different areas of intervention and represented nine professional disciplines.

**Table 3:** Description of characteristics of health professionals

**Health professionals (N = 25)**	**Eastern Townships (n = 9)**	**Montreal (n = 8)**	**Chaudières-Appalaches (n = 8)**
**Gender (F)**	5	7	8

**Living environment (urban)**	7	8	8

**Field of practice**			
Inpatient rehabilitation unit	0	5	5
Acute care hospital	2	3	1
Local community service centre	4	1	1
Day centre	2	0	0
Day hospital	1	1	2
Community organization	1	1	0

**Professional discipline**			
Dietetics	0	1	0
Specialized education	1	0	1
Occupational therapy	1	1	2
Nursing sciences	0	1	2
Social intervention	2	1	1
Neuropsychology	1	0	0
Speech therapy	1	1	0
Physiotherapy/Physical	3	2	2
Rehabilitation therapy			
Psychology	0	1	0

**Experience with stroke clientele**			
Less than 2 years	0	0	2
2–4 years	1	1	0
5–8 years	1	1	2
9 years or more	7	6	4

Finally, 18 health care managers took part in the discussion groups (Table 4). They came from seven different practice areas; two-thirds had clinical experience with stroke patients. Eight of them had over 10 years' management experience (see Table 4).

**Table 4:** Description of characteristics of health care managers

**Health care managers (N = 18)**	**Eastern Townships (n = 7)**	**Montreal (n = 8)**	**Chaudières-Appalaches (n = 3)**
**Gender (F)**	5	7	8

**Living environment(urban)**	6	8	3

**Field of practice:**			
Inpatient rehabilitation unit	1	5	2
Acute care hospital	1	2	0
Local community service centre	1	1	1
Day centre	1	0	0
Day hospital	1	1	1
Community organization	1	0	0
Rehabilitation centre	1	0	0

**Clientele targeted by work:**			
General	3	3	3
Over 65 years	2	3	0
Neurology	2	2	0

**Field of study**:			
Management	3	4	1
Health	6	7	3

**Number of years' management experience:**			
Less than 5 years	2	3	2
5–10 years	1	1	1
More than 10 years	4	4	0

### Needs related to personal factors (capabilities)

Nine categories of capabilities were documented out of a possibility of 10 listed in the DCP model [18]. In fact, only the "breathing capabilities" category was not discussed by participants. The themes that emerged for each category are presented in Table 5. The capabilities that drew the most interest in terms of partially met or unmet needs were those related to behaviour, language, motor activities and sexual relations. The caregivers were especially sensitive to behavioural changes in the patient, which was also the case for the health professionals, though to a lesser extent. The health care managers were particularly sensitive to the needs related to language capabilities, while the patients themselves seemed more affected by partial or unmet needs relating to motor activities, such as walking and gripping ability, as well as more intimate activities such as sexual relations. However, Table 5 shows four categories of capabilities that were not identified as partial or unmet needs by the patients and three that were not identified as such by the health care managers.

**Table 5:** Partially met and unmet needs with respect to personal capabilities

**Capabilities**	**No. of iterations reported by**				**Themes**
	Caregivers	Health Professionals	Healthcare Managers	Patients	
Intellectual capabilities	1	2	0	2	▪ Stimulation (neuropsychology, speech therapy, occupational therapy)
					
Language capabilities	5	5	8	0	▪ Aphasia: Learning to point ▪ Aphasia: Relearning words with pictures ▪ Aphasia: Relearning to write ▪ Aphasia: Communicating on the computer
					
Behaviour capabilities	33	8	1	7	▪ Valuing, security, acceptance, being loved, keeping up to date ▪ Follow-up for depression (psychology) ▪ Follow-up for periods of mourning: agressivity, revolt, frustration, discouragement, anxiety, hope ▪ Changes of role and timetable: sitting, dependence on family for ADL, outings organized differently, driving car
					
Sense and perception capabilities	6	1	1	0	▪ Unilateral-neglect, re-education: reading, eating, dressing ▪ Hypersensitivity on affected side ▪ Spasticity ▪ Pain
					
Motor activity capabilities	3	1	5	10	▪ Learning to walk again, loss of balance, climbing stairs ▪ Physical exercises, stiffness and follow-up ▪ Reeducation of upper limb and follow-up
					
Digestion capabilities	3	3	2	0	▪ Dysphagia, re-educating family: food and swallowing ▪ Discussion (occupational therapist, speech therapist, dietician)
					
Excretion capabilities	3	0	0	0	▪ Enuresis: acceptance and dignity ▪ Support at day centre
					
Reproduction capabilities	2	3	0	10	▪ Expressing sexuality ▪ Availability of information (little discussed by health professionals)
					
Protection and resistance capabilities	0	1	1	1	▪ Pain ▪ Tiredness: car driving and walking long distances

### Needs related to environmental factors

Table 6 presents the themes emerging for each environmental factor of the DCP model, according to the order shown in Figure 1. Four politico-economic factors were documented out of a possible seven: socio-health system (medical care, rehabilitation, social assistance), education system (other types of instruction), public infrastructure and community organizations. Needs related to the political system and governmental structure, the judicial system or the economic system were not reported by any of the participants. One of the two sociocultural factors of the model was explored. Social rules were not addressed by the participants. Finally, some physical elements were discussed.

**Table 6:** Partially met and unmet needs with respect to environmental factors

**Environmental Factors**	**No. of iterations reported by**				**Themes**
	Caregivers	Health Professionals	Healthcare Managers	Patients	
***Political economic factors***					
					
Medical care (socio-health system)	33	30	24	32	▪ Information on existing services ▪ Access to services (delays and disparities) ▪ Follow-up and transfer of files between establishments ▪ Respite services for caregivers: lodging, sitting ▪ Long-term follow-up by CLSC^1^ ▪ Information on medication ▪ Follow-up on taking medication
					
Rehabilitation (socio-health system)	64	63	17	39	*Rehabilitation*: ▪ Multi-disciplinary care for patients: speech therapist, neuropsychologist, psychologist, nutritionist ▪ Evaluation of care dependent on budget and services offered rather than on patient's needs ▪ Personalized approach: length of stay, competency of staff with respect to aphasia, knowledge of patient's file, feeding, alternative therapy, intensity of interventions, services limited in some disciplines (**speech therapy**), attitude of staff with respect to overprotection, staff rotation versus counselling *Adjustment*: ▪ Access to services (delays): assistive technology, adaptation of home and vehicle ▪ Disparities between services offered in different CLSC territories ▪ Obtaining AT and support for care process: AT for the bathroom, clamp, electric bed, portable hoists, AT feeding, grab bars, emergency call button ▪ Home adaptation, support for care process: door frames, bathroom, access ramps, lift on rails, stairs, exiting the home ▪ Vehicle adaptation ▪ Follow-up on attribution of AT and home adaptation ▪ Support and means to find resources in the community (social worker, doctor) ▪ Psychosocial support offered to the family at start and end of stay ▪ Meeting with family at start and end of stay
					
Social assistance (socio-health system)	15	12	10	4	▪ Information on existing services ▪ Discussion group and support for caregivers ▪ Discussion group for patients ▪ Sitting or respite services ▪ Day centre ▪ Temporary lodgings ▪ Support service for meeting with volunteers ▪ Voluntary support and partnership: life project ▪ Who does what: meetings, voluntary work, services, care...
					
Other types of instruction (education system)	14	23	10	19	▪ Direct instruction to caregivers/family by health professionals (bathroom hygiene with AT, practising walking, preventing falls, medication, aphasia, exercises, state of health, nutrition, feeding and nourishment, basic care such as using the toilet) ▪ Integration of caregivers/family *in vivo *during care interventions (gym, therapy, services plan, day hospital) ▪ Prevention of falls (information meeting, video) ▪ *Momentum *for communicating information to patient and caregiver ▪ Education on consequences and impacts at home (preparing for return home) ▪ Education on mourning process ▪ Equip caregivers with tools to find services for the patients, answers to their questions and support resources
					
Public infrastructures	4	2	3	10	▪ Adapted/accessible transportation or paratransit
					
Community organizations	1	9	2	2	▪ Information on existing services, directories ▪ Community services for stroke survivors ▪ Transport by community organizations ▪ Promotion and education by certain community organizations relating to consequences of stroke (values, attitudes)
					
***Sociocultural factors***					
					
Social Network	6	6	1	7	▪ Family: availability and relationships ▪ Friends: social climate
					
***Physical factors***					
					
Architecture; National and regional development	2	0	0	7	▪ Circulation space in public places (walking frame, wheelchair): sidewalks, ramps, stairs ▪ Parking spaces ▪ Rest areas: public benches ▪ Use of doors
					
Technology	0	2	2	6	▪ Access to mobility aids for shopping (wheelchair, walking frame, tripod cane) ▪ Access to special needs equipment (pool, treadmill, rails...)

1 : center of local community services

#### Socio-health system

At the medical care level, all the groups agreed on needs relating to financial and human resources (scarcity of resources), and most participants talked of difficulty accessing the services (delays and disparities) and obtaining follow-up. Partially met or unmet needs were reported at several levels of rehabilitation. For ease of understanding in Table 6, all the interventions aimed at improving a person's potential were classified under "rehabilitation", while those concerning adjustment to the human and physical environment were classified under "adjustment". Adjustment becomes necessary when an individual's rehabilitation has reached a plateau; at that stage the individual's disabilities must be compensated by the human or physical environment [82]. The themes arising from the discussions indicate that rehabilitation is not often personalized to the needs of the patient and that, for reasons of budget and availability of resources, the emphasis is put more on evaluating patients than on rehabilitation activities. Lack of resources in psychology, speech therapy and neuropsychology was also reported. On the issue of adjustment, the caregivers and health professionals exposed a number of problems relating to services for adapting the patient's home or vehicle and with respect to obtaining technical assistance. In addition to the disparities and delays in the various services, there are also persistent problems in the continuity of these services. Furthermore, on the issue of adjustment, not enough support is given to patients and caregivers to identify the resources available to them in the community. Finally, the psychosocial support offered to families who are in the process of rehabilitation is not necessarily available at the right time. To sum up the needs with respect to the socio-health system, *social assistance *emerges as an unmet need that is criticized by many. Access to support groups for caregivers, sitting and respite services and temporary lodging remain unmet needs expressed by many participants.

Regarding the education system, with respect to other types of instruction (not related to academics), there is a pressing need to educate caregivers and to give the instruction at the right time. The need for education was discussed in all the groups and to a greater degree among the health professionals. Insofar as public infrastructure is concerned, only the need for accessible/adapted transportation or paratransit was described as being partially met or unmet. The issue of community resources was hardly raised at all; this appeared to be a little known subject by all the groups of participants. Finally, with respect to support from community organizations, the caregivers and health professionals were the two main groups who expressed concerns about needs in this area.

#### Sociocultural factors

Just one sociocultural factor was discussed: the social network. This aspect remains an unmet need that was identified unanimously by the caregivers, health professionals and patients alike. The main points raised concerned relations with family, friends and organizations promoting awareness in the community.

#### Physical factors

At the development level of the DCP model's physical factors, partial and unmet needs were addressed, if succinctly, in terms of architecture, national and regional development and technology related to medical devices or assistive technology used by people with stroke.

### Needs related to life habits

Table 7 presents all the themes that emerged per life habit according to the Disability Creation Process [18]. Eleven life habit areas were documented out of a possible twelve; only "Education" was not discussed. One life habit was the subject of heated discussions in all the groups: interpersonal relationships. The managers and patients in particular reported various problems persisting in relationships with friends, family members, partners, other patients and in the community. The issue of carrying out personal care at home also emerged as an unmet need and was discussed by all four groups, more especially by the health professionals. The main difficulties lie in dressing, use of the toilet, and personal hygiene. Travelling short and long distances was also reported as a widespread problem, but only by the caregivers and the patients. Eating food and maintaining a healthy body and mind were problematic life habits, mainly for the patients. The health professionals in particular underlined the persistant difficulty in carrying out leisure activities. Finally, the problem of sending and receiving messages was raised by the caregivers and health professionals, with respect to the need for more stimulation in oral and written communication. Finally, four life habits were discussed that could not be compared among the four groups: habits related to housing, taking responsibilities, community life and employment (including volunteer activities).

**Table 7:** Partially met and unmet needs with respect to life habits

**Life habits**	**No. of iterations reported by**				Themes
	Caregivers	Health Professionals	Health Care Managers	Patients	
Nutrition	3	4	0	8	▪ Preparing food ▪ Independent feeding. Feeding oneself ▪ Using kitchen utensils with one hand ▪ Meals-on-wheels service ▪ Diet to be followed ▪ Difficulty swallowing
					
Fitness	1	5	3	9	▪ Enrolling in a group activity requiring use of upper limb ▪ Exercise program offered on discharge ▪ Changing bad habits (smoking, alcohol, eating, exercise)
					
Personal care	4	8	3	2	▪ Dressing ▪ Use of toilet ▪ Personal hygiene
					
Housing	1	0	0	1	▪ Supervision at home and safe movement
					
Mobility	8	0	0	8	▪ Walking long distances ▪ Driving a car, losing a license ▪ Trips outside the home with community organization (volunteers) restricted to bank, doctor ▪ Adapted/accessible transportation (outings, doctor) ▪ Taxi (expensive)
					
Communication	5	6	0	0	▪ Stimulation for communication: reading, speaking
					
Responsibility	0	1	0	1	▪ Change in roles: managing budget, paying bills, doing personal care, going to the bank
					
Interpersonal relationships	4	6	10	9	▪ Visit from friends or family (movement more difficult) ▪ Family relations (arguments, humiliation...) ▪ Intimate and sexual relations difficult in lodgings ▪ Relationship with partner (prevention: separation, divorce) ▪ Resources for meeting people socially ▪ Relationships with other patients
					
Community life	0	1	1	3	▪ Going to church (spiritual life) ▪ Shopping difficult if have to walk (-) and carry bags (-) ▪ Adapted leisure activities ▪ Using banking services
					
Employment and other habits	0	1	2	0	▪ Social integration through leisure, adapted or regular work or volunteer work: find partners outside the health network
					
Recreation	2	8	1	3	▪ Availability of volunteers for adapted/accessible transportation ▪ Open-air activities in wheelchair and adapted vehicle ▪ Community leisure activities organized through day centre ▪ Seeing new areas ▪ Going on travels ▪ Using restaurants

## Discussion

The results show that partially met and unmet needs exist with respect to nine types of personal capabilities, nine environmental factors and the majority of life habits (11 out of 12). In light of these results, what conclusions can we draw from the analysis of the expressed and normative needs of each of the four groups of experts consulted? Furthermore, how can the results obtained improve what we currently know about the rehabilitation needs of people with stroke? Finally, what were the advantages and limitations of using the DCP model for this study?

### Expressed, normative and comparative needs

Half of the expressed needs corresponded to patients' demands and were inherent to activities carrying social importance, such as feeling well, walking, having sexual relations, cooking, keeping fit, visiting friends, driving or using public transport. Few had restarted social activities outside the home. Patients interviewed individually expressed the same opinions as those participating in the focus groups. They would like more access to resources to reduce the burden on caregivers and to be more independent, but they did not mention what kind of resources, except for adapted/accessible transportation or paratransit. It is also possible that the stroke survivors did not specifically mention what kind of resources because they did not know what resources were available. Like the health care managers, they talked less about the nature and type of needs that are unmet or partially met.

The other half of the expressed needs corresponded to requests from caregivers. They said they would like more means with which to facilitate patients' integration at home and in the community. They find it hard to cope with the behavioural problems of stroke sufferers and, like the health professionals, they deplore certain shortcomings in the adjustment and rehabilitation services (Table 6, n = 64 iterations) and the critical need for social assistance and education. This is probably linked to the fact that the persons they were taking care of had more serious disabilities than the patients participating in the focus groups and interviews. The caregivers, like the health professionals, talked more about what should be done and were more specific in how they perceived rehabilitation and social support services than the patients and health care managers.

Normative needs were for the most part reported by the health professionals, whose goal is to provide optimal rehabilitation services according to standard practices in the field, but who also raised concerns relating to intervention type, time restrictions, the institutional environment and the type of disability. They said they have very little time in which to provide the direct and indirect services necessary to ensure complete rehabilitation and to follow up on patients after hospital discharge; they therefore rely on other resources for making the adjustments needed in the home environment (Table 6, n = 63 iterations). The health professionals talked of their frustration in dedicating a large amount of time to evaluating patients and identifying rehabilitation goals but not being able to achieve all those goals. It would therefore seem that health professionals have to make intervention choices more often according to the limitations of the institutional environment in which they work, even if clinical data for their stroke patients show that some of those patients should be given more time and resources. The health professionals said they concentrate their energies on the needs of people with severe disabilities. Patients with only slight or unnoticeable disabilities receive hardly any treatment from them and are referred to other resources.

Comparative needs were mostly reported by the health care managers; they believe it is important that responsibility for patients be properly assured by the teams of professionals in charge of them. This is an attempt to standardise provision. According to the managers, the patients' needs are inextricably linked with those of their families, and in this sense, the managers heavily emphasized the need to offer more instruction to caregivers and to make more support resources available to them and to the patients when they return home. In one of the three groups of managers, the discussion revolved mainly around the availability of services rather than how accessible they were (Table 6, medical care). They suspect that there is a trend among health professionals to want to delegate responsibility for patient care. The managers reported needs centred on means, i.e. available resources (financial, human, physical) and the demand for services for stroke patients and their caregivers (Table 6, rehabilitation). The managers were, of course, aware of the service shortages in certain disciplines (e.g.: speech therapy) and for the pressing need to find solutions for the well-being of patients, but they did not have a lot to say on the subject of making the interventions more personalized to meet the needs of patients and caregivers. Like the patients, the health care managers talked more in terms of availability of resources, and in a more global sense.

### New awareness of rehabilitation needs and contribution of needs classification

By comparing the viewpoints of the various people involved, we can see a division between the two groups of patients/health care managers and health professionals/caregivers. Neither the patients nor the health care managers have to give direct assistance. Patients receiving services or support are more concerned with having lost former significant occupations (e.g. in. Table 7 : Preparing food, walking long distance, driving a car), which is backed by the study of Brandiet et al. [60]. As for the managers, they are primarily concerned with the productivity of their departments: they must oversee the smooth functioning and organization of a service where patients, caregivers and health professionals meet, and they cannot deal with the individual needs of each in any detail. What the patients and the managers have in common is their concern for the how things stand at a given moment, to be able to draw a profile of the situation. It is a completely different scenario for the health care professionals and the caregivers, who have to deal with behaviour that varies from one patient to another and with the limitations imposed by the very specific needs of persons with stroke (e.g. in. Table 5 : Follow-up for periods of mourning: agressivity, revolt, frustration, discouragement, anxiety, hope). In this respect, the needs perceived by health care professionals and caregivers are more centred on how the individual functions in his or her environment and on the intervention methods needed to optimize that person's rehabilitation and adjustment. Even though the individualized approach is seen as the key to successful rehabilitation, it is not often used, according to the health care professionals, the caregivers and the patients. Many times, the caregivers reported the lack of personalization in interventions and education. They were in no way criticizing the skills of the health care professionals, but rather their availability and the momentum behind their interventions.

### New awareness of rehabilitation needs and contribution of DCP model

The partially met and unmet needs (expressed, normative) arising in the various capabilities remain similar to those that have been documented (normative needs) with regard to cognitive problems (intellectual and language capabilities) [14,47-51], perceptual problems (sense and perception, protection and resistance) [47-51,72], affective problems (behaviour) [60-62,72], and motor problems (motor activity) [14,44,45,72]. The needs reported relating to behavioural skills corroborate existing literature with respect to a noticeable decline in functional and psychosocial activities during the first four years after the inpatient rehabilitation period and the fact that a number of rehabilitation interventions have not been completed when the patient is discharged [11,59,71,75]. The patients and caregivers therefore expressed similar needs to those raised by the health care professionals. With respect to digestion, excretion and sexual relations (reproduction capabilities), as far as we are aware there are no points of comparison with literature on this subject.

The partially met and unmet needs that emerged with respect to environmental factors go beyond those that are already known and published in scientific journals. The importance of meeting needs such as preparing for going back home, care to be given, education, information and referral to community support resources [5,38,43,55,60,64-67,72,83] are all issues raised in our study and which are reported under themes linked to the socio-health system and education system under the DCP model. In this respect, the results obtained from our 12 focus groups corroborate the literature: many patients continue to face problems of social participation and many of these intensive post-rehabilitation needs have not been met. The originality of the results of this study lies in the factors explaining the unmet rehabilitation needs. Furthermore, the partially met and unmet needs relating to environmental factors also show that they are far from optimal with respect to assuring the successful participation of patients returning home. In this respect, the financial and human resources, medical care and related services, the type of instruction given to caregivers, the support provided by community organizations, the public infrastructures offering community services and the adjustment and rehabilitation services all reveal considerable shortcomings. The themes that emerge in relation to these factors could constitute means to achieve beneficial solutions for various types of decision-makers, ranging from care services administrators to public administrators in charge of designing public spaces.

Finally, partially met and unmet needs expressed in relation to life habits are concordant with previous studies, but they are more specific than the problems identified of a functional nature (stroke and mobility) [10,60,61] and psychosocial nature [22,40,43,49,52-59,72]. Our study identifies needs associated with eating habits, personal care, housing, travel, communication, consumption of goods and services, as well as fitness of body and mind, taking responsibilities, relationships with others, primary occupation and leisure activities. The sub-themes that emerged suggest ways towards concrete solutions for the various levels of decision-makers.

In short, the DCP model will have made it possible to highlight the partially met and unmet needs relating to environmental factors that were not mentioned in literature. This finding is hardly surprising, for most of the studies already conducted did not concentrate on elderly people's adjustment to their home environments, whether in the home or in the community.

### Strengths and limitations of the study and future research

This study presents a number of strong points with respect to trustworthiness of qualitative research. Certain strategies suggested by Krefting [80] ensure rigor without sacrificing the relevance of the qualitative research. First, to ensure the credibility (truth value) of results, it was developed by researchers in different disciplines involved in the rehabilitation of older adults and considers the views of everyone involved in the process, from the patient to the health care manager. Furthermore, the density of descriptions obtained in the various groups helps us get a clear understanding of the expressed and normative needs put forward. To ensure the transferability (applicability) of the results, the diversity of the sample sought when recruiting by reasoned choice as well as the description of the study area and the participants (n = 72 including 17 patients post-stroke) is detailed enough to allow readers to make evaluations from the possible transfer of data to similar contexts. The study area included three distinct geographic regions (urban, semi-urban and rural), six different fields of practice in rehabilitation and four types of expert groups. Regarding dependability (consistency), each stage of research was documented and various cases were analyzed until a consensus was reached between the researchers. All the focus group participants received a written report summarizing the themes that had emerged in their group discussions for validation. They were then all contacted to check whether they wanted to make any changes or additions to the reports. A few minor changes were made to the summary tables following these validations with participants. The reverse-coding of the transcriptions by the research team and coding validation meetings guaranteed the reliability of the results. Finally, the use of theoretical perspectives (DCP model) for analysis means and the production of data analysis reports for the researchers made it possible to ensure confirmability (neutrality).

The limitations of this study are associated with the research design (descriptive, transversal). The results only take account of the socio-political, socio-cultural and physical environments of three regions of Quebec. Due to the size of the groups, the transferability of the data cannot be completely assured, at least for persons with stroke. Indeed, given the various possible profiles of stroke patients, it is plausible that some needs were not addressed in our study, because we did not have a sufficiently wide representation of patients. There is a certain selection bias inherent to participation in the focus groups or interviews: patients whose cognitive capabilities are more greatly affected or those who have difficulty expressing themselves could not be interviewed. Nevertheless, such patients were, in part, represented by the caregivers.

Other studies are necessary to continue validation of the needs according to the Disability Creation Process framework. The expected results of a far-reaching longitudinal study led by our group will enable us to document more precisely the various degrees and types of disabilities of people with stroke, how they carry out their life habits and the obstacles they come up against in their environment.

## Conclusion

Better knowledge of the needs of people with stroke in accomplishing the activities and social roles they value is essential for improving rehabilitation services, because social participation is recognized as being the ultimate goal of rehabilitation. The presence of handicap situations in areas such as interpersonal relationships, mobility and leisure can isolate the person and foster the development of secondary disabilities if appropriate interventions are not offered. A better knowledge of rehabilitation needs and changes in them after discharge from formal services will improve coordination of these services and develop other services to address needs that are not currently being met, in the aim of maintaining the population active in social roles. In this respect, the results show that needs persist after patients are discharged, relating to nine capabilities in patients, nine environmentally related factors and eleven life habits. Close caregivers and health professionals identify more unmet needs and put the emphasis on the importance of making the rehabilitation process more personalized. The patients and health care managers have a more global vision of rehabilitation needs and put greater emphasis on available resources. To encourage better social participation of elderly people with stroke when going back at home, the study suggests that more should be done to meet the needs relating to capabilities, the patient's environment and life habits. Reducing the obstacles in the socio-politico-economic environment becomes essential for assuring a more personalized approach to rehabilitation and better instruction for caregivers.

## Competing interests

The authors declare that they have no competing interests.

## Authors' contributions

CV conceived the design of the study, participated in the data analysis, and outlined and drafted the manuscript. ID managed the database, performed the data analysis and commented the manuscript. LR conceived design the study, participated in the data analysis and commented the manuscript. JR conceived design the study, participated in the data analysis and commented the manuscript. ChV formed the focus groups and conducted the interviews, performed the data analysis and commented the manuscript. JD was the principal investigator, submitted the research protocol for its financing, conceived the design of the study and the coordination of all the research meetings, participated in the data analysis and commented the manuscript. These authors contributed equally to this work. LRT participated in the design of the present study, formed the focus groups and commented the manuscript. All authors read and approved the final manuscript.

## Pre-publication history

The pre-publication history for this paper can be accessed here:



## References

[B1] American Heart Association Statistics Committee and Stroke Statistics Subcommittee (2006). Heart Disease and Stroke Statistics – 2006 Update. Circulation.

[B2] U.S Department of Health and Human Service (1995). Public Health Service, Quick Reference Guide for Clinicians, no 16, Rockville, USA.

[B3] Kaste M, Palomäki H, Sarna S (1995). Where and how should elderly stroke patients be treated? A randomized trial. Stroke.

[B4] Osberg JS, McGuinnis GE, DeJong G, Seward ML, Germaine J (1988). Long term utilization and charges among post-rehabilitation stroke patients. Am J Phys Med Rehabil.

[B5] O'Connell B, Hanna B, Penney W, Pearce J, Owen M, Warelow P (2001). Recovery after stroke: A qualitative perspective. J Qual Clin Practice.

[B6] Angeleri F, Angeleri VA, Foschi N, Giaquinto S, Nolfe G, Saginario A, Signorino M (1997). Depression after a stroke: an investigation through catamnesis. J Clin Psychiatry.

[B7] De Haan R (1993). Measuring Quality of Life in Stroke. Stroke.

[B8] Neimi ML, Laaksone R, Kotila M, Waltimo O (1988). Quality of life, 4 years after stroke. Stroke.

[B9] Rosemarie BK (1996). Quality of life after Stroke. Stroke.

[B10] Pound P, Gompertz P, Ebrahim S (1998). A patient-centered study of the consequences of stroke. Clin Rehabil.

[B11] Gladman JRF, Lincoln NB, Barer DH (1993). A randomized controlled trial of domiciliary and hospital-based rehabilitation for stroke patients after discharge from hospital. J Neurol Neurosurg Psychiatry.

[B12] Mayo NE, Wood-Dauphinee S, Ahmed S, Gordon C, Higgins J, Mcewen S, Salbach N (1999). Disablement following stroke. Disabil Rehabil.

[B13] Chuang KY, Wu SC, Ma AH, Chen YH, Wu CL (2005). Identifying factors associated with hospital readmissions among stroke patients in Taipei. J Nurs Res.

[B14] Lincoln NB, Gladman JRF, Berman P, Luther A, Challen K (1998). Rehabilitation needs of community stroke patients. Disabil Rehabil.

[B15] Edwards DF, Hahn MG, Baum CM, Perlmutter MS, Sheedy C, Dromerick AW (2006). Screening patients with stroke for rehabilitations needs: validation of the post-stroke rehabilitation guidelines. Neurorehabil Neural Repair.

[B16] Bates B, Choi JY, Duncan PW, Glasberg JJ, Graham GD, Katz RC, Lamberty K, Reker D, Zorowitz R (2005). Veterans Affairs/Department of Defense Clinical Practice Guideline for the Management of Adult Stroke Rehabilitation Care: executive summary. Stroke.

[B17] Fougeyrollas P, Noreau L, Bergeron H, Cloutier R, Dion SA, St-Michel G (1998). Social consequences of long term impairments and disabilities: conceptual approach and assessment of handicap. Int J Rehabil Res.

[B18] Fougeyrollas P, Cloutier R, Bergeron H, Côté J, St-Michel G (1999). The Quebec Classification: Disability Creation Process.

[B19] Martin BJ, Yip B, Hearty M, Marletta S, Hill R (2002). Outcome, functional recovery and unmet needs following acute stroke. Experience of patient follow up at 6 to 9 months in a newly established stroke service. Scott Med J.

[B20] Bonita R, Solomon N, Broad JB (1997). Prevalence of stroke and stroke related disability. Stroke.

[B21] Dombovy ML, Basford JR, Whisnant JP, Bergstralh EJ (1987). Disability and use of rehabilitation services following stroke in Rochester, Minnesota, 1975–1979. Stroke.

[B22] Burton CR (2000). Living with stroke: a phenomenological study. J Adv Nurs.

[B23] Cloutier-Fischer DS (2005). Different strokes: need for help among stroke-affected persons in British Columbia. Can J Public Health.

[B24] Mayo NE, Wood-Dauphinee S, Côté R, Durcan L, Carlton J (2002). Activity, participation, and quality of life 6 months poststroke. Arch Phys Med Rehabil.

[B25] Liu C, Thompson AJ, Playford ED (2004). Patient dissatisfaction: Insights into the rehabilitation process. J Neurol.

[B26] Lebel P, Arcand M, Hébert R (1997). Services gérontologiques et gériatriques. Précis pratique de gériatrie.

[B27] Trahan L, Bélanger L, Bolduc M (1993). Une évaluation de la prestation de services dans les CLSC et les centres hospitaliers Pour des services de qualité aux personnes âgées en perte d'autonomie.

[B28] Shultz AA (1997). Identification of needs of and utilisation of resources by rural and urban elders after hospital discharge to the home. Public Health Nurs.

[B29] Bradshaw J, Gilbert N, Specht H (1977). The concept of social need. Planning for social welfare, issues, models and tasks.

[B30] Pineault R, Daveluy C (1986). La planification de la santé.

[B31] Béland F (1987). Identifying profiles of service requirements in a non-institutionalised elderly population. J Chronic Dis.

[B32] Liu KPY, Chan CCH, Chan F (2005). Would discussion on patients' needs add value to the rehabilitation process?. Int J Rehabil Res.

[B33] Williams J, Lyons B, Rowland D (1997). Unmet long-term care needs of elderly people in the community: a review of the literature. Home Health Care Serv Q.

[B34] Garrett D, Cowdell F (2005). Information needs of patients and carers following stroke. Nursing older people.

[B35] Belciug MP (2006). Concerns and anticipated challenges of family caregivers following participation in the neuropsychological feedback of stroke patients. Int J Rehabil Res.

[B36] Lui MHL, Mackenzie AE (1999). Chinese elderly patients' perceptions of their rehabilitation needs following a stroke. J Adv Nurs.

[B37] Van Heugten C, Visser-Meily A, Post M, Lindeman E (2006). Care for carers of stroke patients: evidence-based clinical practice guidelines. J Rehabil Med.

[B38] Tooth L, Hoffmann T (2004). Patient Perceptions of the Quality of Information Provided in a Hospital Stroke Rehabilitation Unit. British Journal of Occupational Therapy.

[B39] Monaghan J, Channell K, McDowell D, Sharma AK (2005). Improving patient and carer communication, multidisciplinary team working and goal-setting in stroke rehabilitation. Clin Rehabil.

[B40] Ministère des travaux publics et services gouvernementaux (1999). Les défis d'une société canadienne vieillissante 1999 et après.

[B41] Garant L (1994). Synthèse d'un programme d'évaluation sur la réponse aux besoins de longue durée des personnes âgées ayant des limitations fonctionnelles.

[B42] Association des hôpitaux du Québec et fédération de la réadaptation en déficience physique du Québec (1997). Centres hospitaliers et établissements de réadaptation : partenaires pour la complémentarité des services de réadaptation Une vision et des actions intégrées.

[B43] King RB, Semik PE (2006). Stroke caregiving: Difficult Times, Resources Use, and Needs During the First 2 Years. J Gerontol Nurs.

[B44] Heath GW, Fenton PH (1997). Physical activities among persons with disabilities. A public health perspective. Exerc Sport Sci Rev.

[B45] Kumlien S, Axelsson K, Ljunggren G, Winblad B (1999). Stroke patients ready for discharge from acute care: a multidimensional assessment of functions and further care. Disabil Rehabil.

[B46] Boter H, Rinkel GJE, de Haan RJ (2004). Outreach nurse support after stroke: a descriptive study on patients' and carers' needs, and applied nursing interventions. Clin Rehabil.

[B47] Association québécoise des personnes aphasiques (1997). Guide d'information et de référence sur l'aphasie S'informer pour mieux agir.

[B48] Desmond DW, Moroney JT, Sano M, Stern Y (1996). Recovery of cognitive function after stroke. Stroke.

[B49] Grimby G, Andrén E, Daving Y, Wright B (1998). Dependence and perceived difficulty in daily activities in community living stroke survivors 2 years after stroke. A study of instrumental structures. Stroke.

[B50] Le Dorze G, Généreux S, Laporte D, Navennec C, Brassard C, Daigle MA, Hubert M (1998). Les déterminants individuels et sociaux de la réinsertion professionnelle des personnes aphasiques Rapport final (DEIP-1054).

[B51] Tatemichi TK, Desmond DW, Stern Y, Paik M, Sano M, Bagiella E (1994). Cognitive impairment after stroke: frequency patterns, and relationship to functional abilities. JNeurol Neurosurg Psychiatry.

[B52] Bisset AF, Macduff C, Chesson R, Maitland J (1997). Stroke services in general practice. Are they satisfactory?. Br J Gen Pract.

[B53] Feibel JH, Springer CJ (1982). Depression and failure to resume social activities after stroke. Arch Phys Med Rehabil.

[B54] Forer SK, Miller LS (1980). Rehabilitation outcome: comparative analysis of different patient types. Arch Phys Med Rehabil.

[B55] Gauthier L (1995). Comprendre le processus de fardeau subjectif. Master thesis.

[B56] Koenig HG, Kuchibhatla M (1999). Use of health services by medically ill depressed elderly patients after hospital discharge. Am J Geriatr Psychiatry.

[B57] Schmidt S, Herman LM, Koenig P, Leuze M, Monahan M, Stubbers RW (1986). Status of stroke patients: a community assessment. Arch Phys Med Rehabil.

[B58] Viitanen M, Fugl-Meyer KS, Bernspang B, Fugl-Meyer AR (1988). Life satisfaction in long-term survivors after stroke. Scand J Rehabil Med.

[B59] Young J, Forster A (1992). The Bradford Community Stroke Trial: results at 6 months. Br Med J.

[B60] Brandriet LM, Lyons M, Bentley J (1994). Perceived needs of poststroke following termination. Nurs Health Care.

[B61] Lofgren B, Nyberg L, Mattsson M, Gustafson Y (1999). Three years after in-patient stroke rehabilitation: A follow-up study. Cerebrovasc Dis.

[B62] Soderback I, Ekholm J, Caneman G (1991). Impairment/function and disability/activity 3 years after cerebrovascular incident or brain trauma: a rehabilitation and occupational therapy view. Int Disabil Stud.

[B63] Lewinter M, Mikkelsen S (1995). Patients' experience of rehabilitation after stroke. Disabil Rehabil.

[B64] Pierce LL, Gordon M, Steiner V (2004). Families dealing with stroke desire information about self-care needs. Rehabil Nurs.

[B65] Zwygart-Stauffacher M, Lindquist R, Savick K (2000). Development of health care delivery systems that are sensitive to the needs of stroke survivors and their caregivers. Nurs Adm Q.

[B66] Smith LN, Lawrence M, Kerr SM, Langhorne P, Lees KR (2004). Informal carers' experience of caring for stroke survivors. J Adv Nurs.

[B67] Cook AM, Pierce LL, Hicks B, Steiner V (2006). Self-care needs of caregivers dealing with stroke. J Neurosci Nurs.

[B68] Sit JW, Wong TK, Clinton M, Li LS, Fong YM (2004). Stroke care in the home: the impact of social support on the general health family caregivers. J Clin Nurs.

[B69] Grimaud O, Clappier P, Denis M, Riou F (2005). [A qualitative study for identifying determinants of the quality of stroke patient referral.]. Rev Epidemiol Sante Publique.

[B70] Desnoyers D, Lagacé C (1995). La réadaptation: tendances et perspectives Synthèse d'une recension d'écrits.

[B71] Dam M, Tonin P, Casson S, Ermani M, Pizzolato G, Iaia V, Battistin L (1993). The effects of long-term rehabilitation therapy on poststroke hemiplegic patients. Stroke.

[B72] Hare R, Rogers H, Lester H, McManus R, Mant J (2006). What do stroke patients and their carers want from community services?. Fam Pract.

[B73] Evans RL, Northwood LK (1983). Social support needs in adjustment to stroke. Arch Phys Med Rehabil.

[B74] Widen-Holmqvist L, de Pedro-Cuesta J, Holm M, Sandstrom B, Hellblom A, Stawiarz L, Bach-y-Rita P (1993). Stroke rehabilitation in Stockholm. Basis for late intervention in patients living at home. Scand J Rehabil Med.

[B75] Lindmark B, Hamrin E (1995). A five-year follow-up of stroke survivors: motor function and activities of daily living. Clin Rehabil.

[B76] Talbot LR, Viscogliosi C, Desrosiers J, Vincent C, Rousseau J, Robichaud L (2004). Identification of rehabilitation needs after a stroke: an exploratory study. Health Qual Life Outcomes.

[B77] Anderson S, Marlett NJ (2004). Communication in stroke: the overlooked rehabilitation tool. Age Ageing.

[B78] Morgan DL, Krueger RA, King JA (1998). Focus Group Kit.

[B79] Laperrière A, Poupart J, Deslauriers JP, Groulx LH, Laperrière A, Mayers R, Pires AP (1997). Critères de scientificité des recherches qualitatives. La recherche qualitative: Enjeux épistémologiques et méthologiques.

[B80] Krefting L (1991). Rigor in qualitative research: The assessment of trustworthiness. Am J Occup Ther.

[B81] Institut de la statistique View of Regions (Quebec). http://www.stat.gouv.qc.ca/regions/profils/region_00/region_00_an.htm.

[B82] Laboratoire d'informatique et de terminologie de la réadaptation et de l'intégration sociale du Centre François-Charon et Office de la langue française (1995). Dictionnaire de la réadaptation Tome 1: Termes techniques d'évaluation.

[B83] Wilma M, Hopman WL, Verner J (2003). Quality of life during and after inpatient stroke rehabilitation. Stroke.

